# Increasing signal processing sophistication in the calculation of the respiratory modulation of the photoplethysmogram (DPOP)

**DOI:** 10.1007/s10877-014-9613-3

**Published:** 2014-09-11

**Authors:** Paul S. Addison, Rui Wang, Alberto A. Uribe, Sergio D. Bergese

**Affiliations:** 1 Advanced Research Group, Covidien Respiratory and Monitoring Solutions, The Technopole Centre, Edinburgh, EH26 0PJ Scotland, UK; 2 Department of Anesthesiology, The Ohio State University Wexner Medical Center, Columbus, OH USA; 3 Department of Neurological Surgery, The Ohio State University Wexner Medical Center, Columbus, OH USA

**Keywords:** Hemodynamic monitoring, Fluid responsiveness, Pulse oximetry, DPOP, PPV

## Abstract

DPOP (∆POP or Delta-POP) is a non-invasive parameter which measures the strength of respiratory modulations present in the pulse oximetry photoplethysmogram (pleth) waveform. It has been proposed as a non-invasive surrogate parameter for pulse pressure variation (PPV) used in the prediction of the response to volume expansion in hypovolemic patients. Many groups have reported on the DPOP parameter and its correlation with PPV using various semi-automated algorithmic implementations. The study reported here demonstrates the performance gains made by adding increasingly sophisticated signal processing components to a fully automated DPOP algorithm. A DPOP algorithm was coded and its performance systematically enhanced through a series of code module alterations and additions. Each algorithm iteration was tested on data from 20 mechanically ventilated OR patients. Correlation coefficients and ROC curve statistics were computed at each stage. For the purposes of the analysis we split the data into a manually selected ‘stable’ region subset of the data containing relatively noise free segments and a ‘global’ set incorporating the whole data record. Performance gains were measured in terms of correlation against PPV measurements in OR patients undergoing controlled mechanical ventilation. Through increasingly advanced pre-processing and post-processing enhancements to the algorithm, the correlation coefficient between DPOP and PPV improved from a baseline value of R = 0.347 to R = 0.852 for the stable data set, and, correspondingly, R = 0.225 to R = 0.728 for the more challenging global data set. Marked gains in algorithm performance are achievable for manually selected stable regions of the signals using relatively simple algorithm enhancements. Significant additional algorithm enhancements, including a correction for low perfusion values, were required before similar gains were realised for the more challenging global data set.

## Introduction

**Fig. 1:**
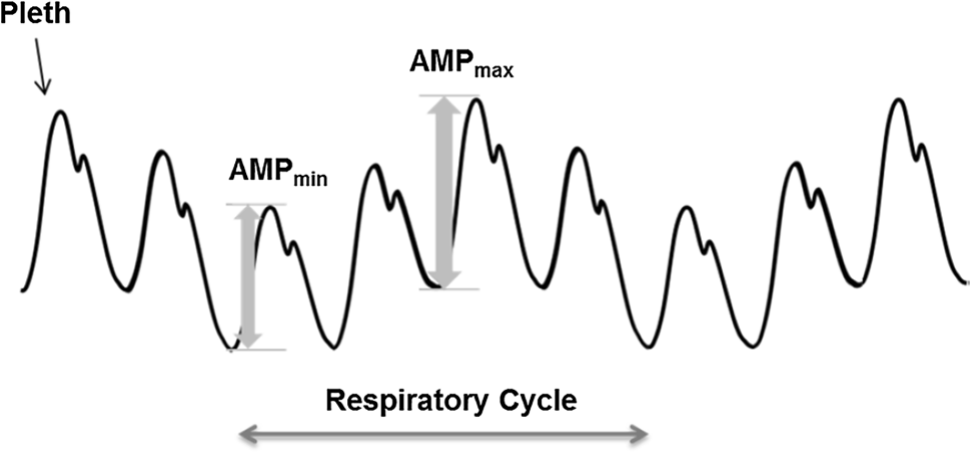
Deriving ∆POP from the Pleth

Volume expansion is commonly used for the critically ill patient to optimize hemodynamic status. Fluid is administered with the expectation that it will increase cardiac preload and cardiac output significantly; however, the response may be variable. Respiratory variation in stroke volume (SVV) allows the clinician to determine where on the Frank-Starling curve the patient’s hemodynamic system is operating. Respiratory modulations in the arterial blood pressure waveform are also known to be a good indicator of likely response to fluid loading in the mechanically ventilated patient [1]. The use of this pulse pressure variation (PPV) parameter to indicate the volemic status of a patient is increasingly widespread in practice, and has therefore been the focus of much attention in this area [2]. DPOP is a non-invasive parameter which measures the strength of respiratory modulations present in the pulse oximetry photoplethysmograph (‘POP’ or ‘pleth’) waveform. It has been proposed as a non-invasive alternative to PPV with many studies showing favourable correlation between the two parameters [3–9, 24]. Cannesson et al. [10] defined the parameter as

1 $$\text{DPOP} \, = \, \left( {\text{AMP}_{\hbox{max} } - \, \text{AMP}_{\hbox{min} } } \right) \, / \, \left( {\left( {\text{AMP}_{\hbox{max} } + \, \text{AMP}_{\hbox{min} } } \right) \, / \, 2} \right)$$

where AMP_max_ and AMP_min_ are the maximum and minimum amplitudes of the cardiac pulse waveforms in the pleth during a respiratory cycle. These are illustrated in Fig. 1, where the cardiac pulse component of the pleth is shown being modulated by respiratory activity.

The development of a fully-automated algorithm capable of coping with the extremes of data characteristics in the clinical environment requires significant processing. Generally such an algorithm consists of three parts [11]: (1) pre-processing, where the raw pleth is manipulated prior to the computation of DPOP; (2) processing, where the computation of the DPOP value according to Eq. (1) is carried out; and, (3) post-processing where the current computed value of DPOP is further processed. These processing steps involve: filtering of the raw signal, assessment of its quality, removal of irregular pulse waveforms, identification and removal of outlying data points, smoothing and, finally, incorporation of the most recently calculated value within the reported value. The latter step may include an average of a number of previous points weighted by temporal relevance and the quality of the data before updating the displayed value to the clinician. The aim of the present study reported here was to demonstrate the performance gains made by adding increasingly sophisticated signal processing components to the DPOP algorithm. The performance gains are measured in terms of a progressively enhanced correlation against PPV measurements in OR patients undergoing controlled mechanical ventilation (Table 1).

**Table 1:** Details of signal processing runs

Run	R_s_	P	Sens	Spec	YI	AUC	R_s-_med	10 % R_s_	90 % R_s_
(a) Stable region data results									
1	0.347	<0.01	0.808	0.751	0.560	0.858	0.355	0.233	0.532
2	0.521	<0.01	0.831	0.729	0.560	0.859	0.522	0.352	0.690
3	0.651	<0.01	0.833	0.712	0.545	0.849	0.645	0.483	0.761
4	0.691	<0.01	0.828	0.743	0.571	0.868	0.685	0.535	0.791
5	0.758	<0.01	0.863	0.741	0.604	0.890	0.753	0.587	0.851
6	0.817	<0.01	0.876	0.713	0.589	0.888	0.813	0.679	0.892
7	0.844	<0.01	0.892	0.697	0.589	0.881	0.842	0.718	0.911
8	0.826	<0.01	0.882	0.709	0.591	0.881	0.823	0.710	0.897
9	0.826	<0.01	0.858	0.699	0.557	0.877	0.824	0.712	0.897
10	0.852	<0.01	0.892	0.819	0.711	0.917	0.848	0.803	0.889

Run	R_g_	P	Sens	Spec	YI	AUC	R_g_med	10 % R_s_	90 % R_g_
(b) Global data results									
1	0.225	<0.01	0.773	0.562	0.335	0.713	0.228	0.144	0.308
2	0.221	<0.01	0.749	0.561	0.311	0.697	0.228	0.154	0.303
3	0.261	<0.01	0.761	0.542	0.303	0.688	0.269	0.165	0.362
4	0.274	<0.01	0.797	0.545	0.342	0.699	0.280	0.181	0.372
5	0.311	<0.01	0.811	0.532	0.343	0.700	0.318	0.210	0.414
6	0.326	<0.01	0.771	0.536	0.306	0.691	0.338	0.221	0.436
7	0.350	<0.01	0.876	0.537	0.413	0.716	0.362	0.247	0.459
8	0.468	<0.01	0.836	0.600	0.435	0.734	0.464	0.279	0.609
9	0.467	<0.01	0.810	0.609	0.419	0.733	0.463	0.273	0.610
10	0.728	<0.01	0.834	0.756	0.590	0.863	0.724	0.559	0.808

Subscripts ‘s’ and ‘g’ refer to stable and global data results respectively

## Methods

### Patients

With institutional review board approval and written informed consent, a convenience sample of adult patients was enrolled at the Ohio State University Wexner Medical Center. Patients requiring the placement of an intra-arterial line who have been scheduled to undergo elective surgery or required surgical intensive care unit (SICU) admission were enrolled in the study. No specific disease states or pathophysiologic conditions were targeted during enrolment. Exclusion criteria were: (1) currently participating in or has participated in an investigational drug study within 7 days of enrolment, (2) known severe contact allergies, (3) existing health conditions preventing proper sensor application, and (4) vulnerable groups.

### Data acquisition

Each patient was fitted with a finger sensor (Nellcor OxiMax Max-A, Covidien, Boulder CO) as per the sensor’s device labelling. The sensor was connected to a custom data-recording box that contained a Nellcor OEM pulse oximeter of the same type found in the commercially available N-600x monitor (Nell-1 board, Covidien, Boulder CO). The blood pressure signal from an intra-arterial blood pressure monitor (Solar 8000, by GE-Marquette) was also recorded. A synchronized acquisition of the pulse oximeter and arterial pressure signals was performed during the whole procedure and saved to the laptop for later analysis.

The resulting OR data set comprised 36 patient records where pleth and arterial line waveforms were collected simultaneously. 16 data sets were excluded from analysis for a variety of reasons, including: the absence of, or missing, information in the case report form (CRF); absence of a blood pressure waveform recording; absence of a pleth waveform recording; presence of an arrhythmia; corrupted data files; and pleth data with artefacts due to BP cuff inflations on the same arm as the oximeter probe. The remaining 20 subjects had a mean length of data record of 115 min, with the shortest recording of 43 min and longest recording of 204 min.

**Fig. 2:**
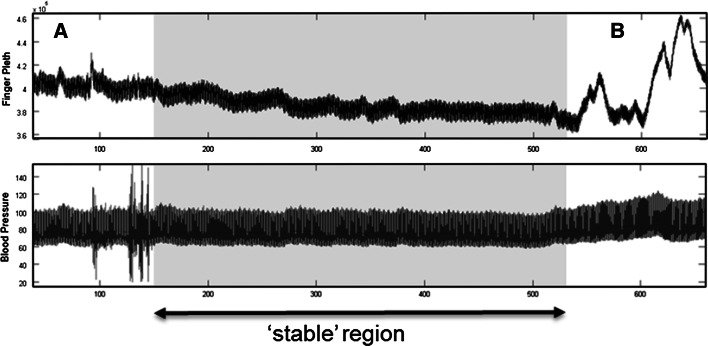
Selection of a stable region (*top* finger pleth, *bottom* arterial BP)

The collected signals were further sub-divided into two distinct data sets for use in the analysis: (1) a stable region data set, and (2) a global data set. The “stable data set” corresponds to a few minutes of high quality signal segments manually selected from within the post-induction, pre-incision period, where only general anaesthetic drugs had been administered (i.e. no vasoactive drugs), and where the pleth and BP signals were deemed artefact free. The stable data set is intended to provide a comparison to results from studies based on manually selected, high signal quality regions often reported in the literature. The identification of the stable region was performed by eye. An example of a stable region selected for analysis is shown shaded in Fig. 2 where the artefact in the signals within regions ‘A’ and ‘B’ caused the exclusion of these parts of the signals from the stable region. The “global data set” contains the entire data record of the patient, i.e. including sections such as A and B in Fig. 2, and is indicative of all data encountered by a commercial device in practice including artefact due to movement, large blood pressure changes, drug infusions, incisions, vasomotion, etc. As such, it provides a much tougher test for an automated algorithm which has to make decisions on signal quality and optimize the reported parameter accordingly via advanced signal processing measures.

### Analysis

**Fig. 3:**
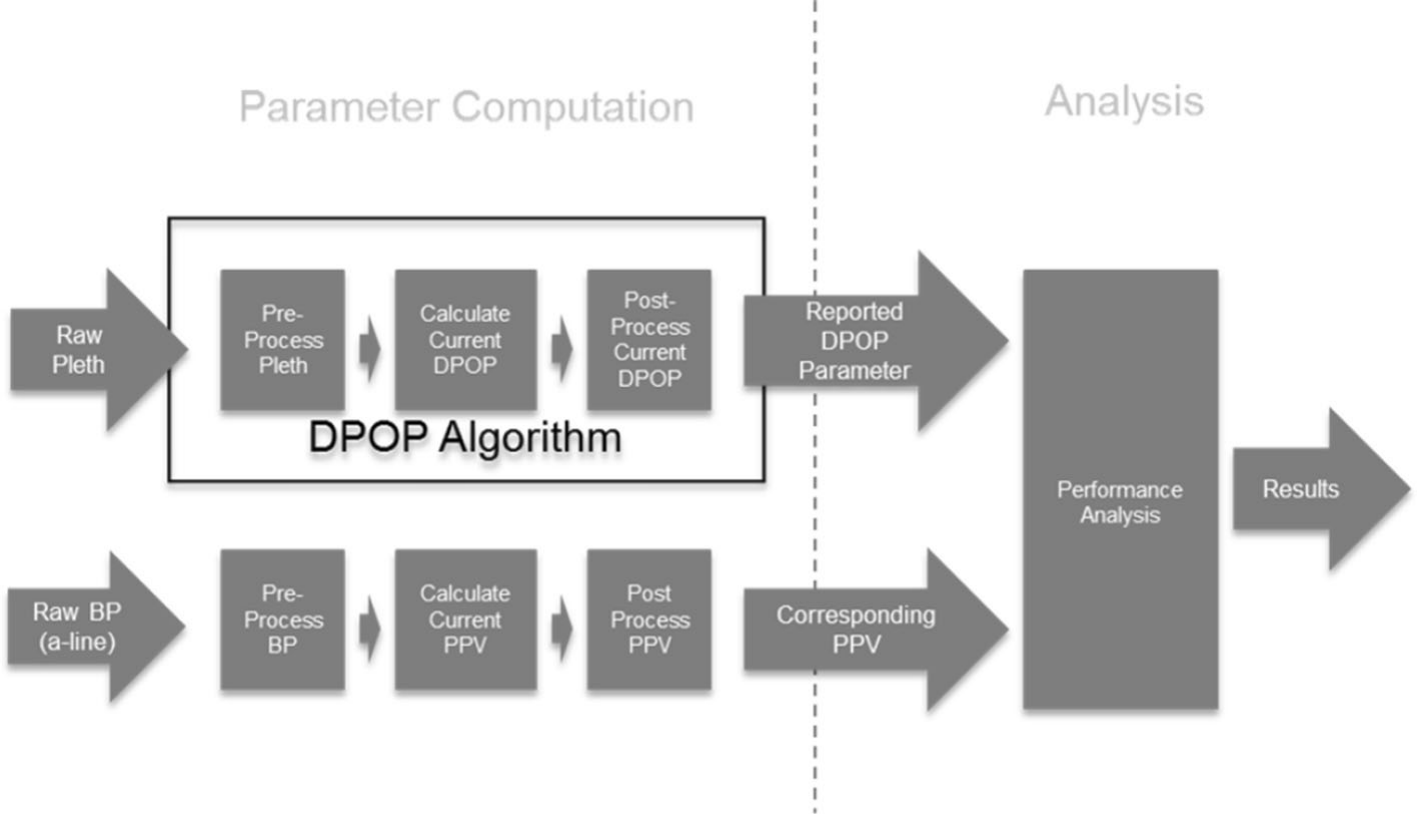
Schematic overview of the algorithm development infrastructure

An algorithm development infrastructure was set up to aid the rapid development and refinement of the parameter where, during each iteration, the candidate DPOP algorithm may be modified according to previous performance characteristics. A schematic of the algorithm development infrastructure is shown in Fig. 3. In this way, various controlling parameters may be examined both independently of each other and in a combined fashion. This parametric analysis allows for the rapid examination of candidate code changes relating to potential improvements to the algorithm. A series of 10 runs was conducted using the patient data sets where increasingly sophisticated processing elements were incorporated within the DPOP algorithm. Note that the PPV calculation was performed using the same processing approach as DPOP during each of the runs, e.g. as buffer lengths and filter characteristics were altered for the DPOP calculation these were matched in the calculation of PPV. These are described in more detail in the results section together with the corresponding incremental improvement in performance.

The performance analysis of the DPOP parameter against the PPV signal involves computing statistics that describe quantitatively their correlation. A linear least square regression line for DPOP versus PPV was plotted. The Pearson correlation coefficient, R, was used to describe how well DPOP fitted the linear relationship with PPV. The statistical significance (*p* value) of R was also calculated. Bootstrapping was used to provide non-parametric accuracy statistics for the computed correlation coefficients: 10th and 90th percentile error bars were determined based on a 1,000-run iteration, random per-subject replacement of the data. Receiver operator characteristic (ROC) curves were also computed, by determining the sensitivity and specificity pairs over a range of DPOP thresholds, together with the corresponding area under the curve (AUC) values. These correspond to the hypothetical substitution of DPOP for PPV achieved by setting a fixed threshold for PPV of 13 % (as used in several studies for indicating the boundary between non-responsive and responsive patients, e.g. Poli de Figueiredo et al. [12], Cannesson et al. [10] Natalini et al. [13], Landsverk et al. [14], Westphal et al. [7] ). We also determined optimal sensitivity/specificity pairs using the pre-defined criterion of maximising the Youden index (sensitivity + (specificity − 1)) [8, 13, 15]. Note that in practice we prefer the use of the R statistic over AUC’s in driving our analysis of performance as it is generally more sensitive to outliers.

## Results

**Fig. 4:**
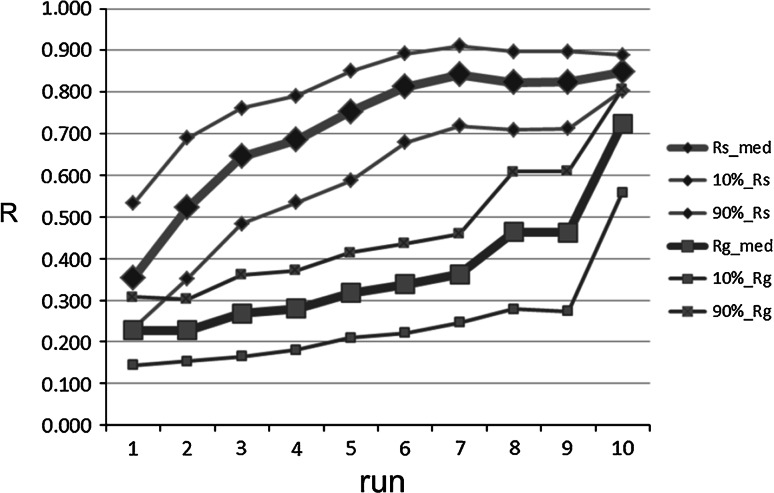
Correlation coefficients for each run. Note that the median values displayed in this graph obtained from 1,000 iteration bootstrapping are equivalent (within 2 decimal places) to the single run values derived through the whole data analysis shown in subsequent figures. Subscripts ‘s’ and ‘g’ relate to the stable and global regions respectively

A series of 10 runs was conducted comprising increasingly sophisticated processing elements incorporated within the algorithm. An overview plot of the performance over the series of runs is provided in Fig. 4 based on the R statistic. This plot highlights the trending over the runs and allows the reader to set in context the incremental performance gain made at each step described below in relation to overall gains. The rest of this section discusses each signal processing improvement in turn and its effect on performance. Note that the values of the parameters associated with each incremental step were determined through parametric analyses carried out to determine the optimal values.

### Run 1: basic run

**Fig. 5:**
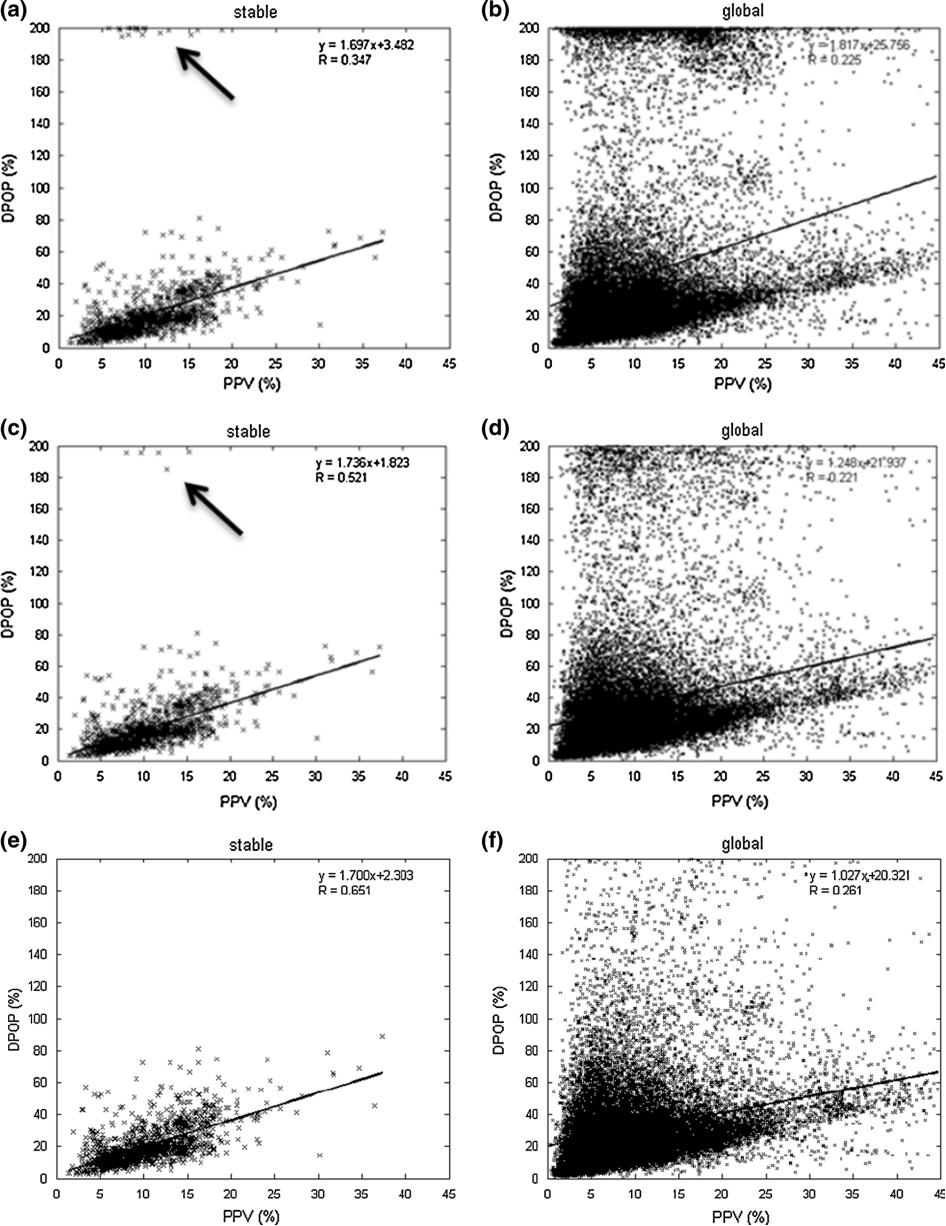
Correlation Plots for runs 1 to 3 **a** Run 1 stable region **b** Run 1 global region** c** Run 2 stable region **d** Run 2 global region **e** Run 3 stable region** f** Run 3 global region

**Fig. 6:**
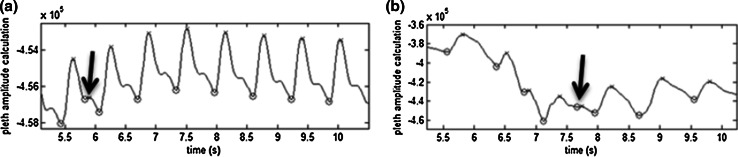
Example of signal erroneous fiducial detections run 1. **a** Dicrotic notch error, **b** excessive baseline error

For this first run, a basic algorithm was implemented which employs a signal turning point detector to determine the minimum and maximum points on each cardiac pulse waveform in the pleth. The beat amplitude was then computed as the difference between these two fiducial points and used in the calculation of DPOP. However, in practice a number of local minima and maxima are present on many of the pulses due to strong dicrotic notches and/or various types of signal noise. These erroneous beat maxima and minima must be removed from each pulse prior to the determination of its true amplitude. In Run 1, this was achieved through a relatively simple method which: (1) locates all peaks on the pleth; (2) removes all smaller peaks occurring in close proximity (<0.35 s) to a nearby larger peak; and (3) searches for the minimum point between peaks (to find the beat minimum point between consecutive beat maxima). DPOP is computed from the resulting pulse amplitudes over a fixed 10 s ‘analysis window’ (large enough to include the expected range of respiratory periods). Figure 5a, b show the correlation plots between PPV and DPOP for both stable and global regions for Run 1. These plots both exhibit relatively low values of Pearson correlation coefficient of R = 0.347 and 0.225 respectively. We can see from the figure that a number of outliers appear near DPOP = 200 % as this relatively simple algorithm implementation is not robust enough to cleanly delineate every whole beat in the data set. In fact, it sometimes wrongly identifies the local minimum and maximum at the dicrotic notch as a separate peak. This amplitude of the dicrotic notch may be erroneously chosen as the minimum pulse amplitude. This has a near zero value compared to the maximum pulse amplitude and hence, through equation (1), the maximum amplitude is divided by approximately half of itself, resulting in a computed value near 200 %. In addition to dicrotic notches, arrhythmic beats and excessive baseline shifts can cause distortion of the pleth pulse leading to misidentified fiducial points and hence errors in the computed DPOP values. This kind of interference occurs frequently in practice and is the cause of much of the spread of data in the global plot. Figure 6 contains two examples of these types of distortions from within the current data set.

### Run 2 and Run 3: pre-filtering of the pleth

In order to mitigate the effects of identifying multiple points per beat, the pleth was pre-processed by low pass filtering the raw signal prior to the calculation of DPOP using a fixed low pass filter (3rd order Butterworth filter with cut-off frequency *f*_*c*_ = 2.83 Hz). This resulted in a marked improvement in the stable region results from Run 1 to Run 2 of R = 0.347 to R = 0.521. A further improvement, to R = 0.651, was achieved in Run 3 where a more flexible adaptive filter was implemented based on the heart rate (set at a cut-off frequency of 1.2*HR/60 Hz, where HR is the heart rate in beats per minute). The global region results improved only slightly over the first three runs from 0.225 to 0.261. These correlation coefficients are markedly smaller than those for the stable regions due to the substantially noisier nature of the extended data set, which include movement, large blood pressure changes, drug infusions, incisions, vasomotion, etc. These are effects that require more than simple filtering of the data to deal with. We can see by comparing the plots for Runs 1 to 3 in Fig. 5 that by Run 3 the pre-processing has removed all outliers at around 200 % for the stable runs and a significant proportion of these outliers for the global region runs.

### Run 4: alternative fiducial detection methods using the derivative pleth

This run corresponds to a method based on identifying fiducial points using the derivative of the pleth. For Run 4, fiducial points of the signal were first identified in the derivative pleth and then mapped back to the original signal where the amplitude information is calculated. The original pleth is first filtered using a band pass filter to smooth out the dicrotic notches before the derivative is computed. For this run an optimum non-zero threshold was obtained empirically every 5 s and used to separate each pulse. A non-zero threshold allows the dominant gradient associated with the pulse systolic rise to be located more easily. The peaks of the derivative are then used to locate and separate the pulses in the time domain. Once this has been carried out, the local pulse maxima and minima are found and used to determine pulse amplitudes. This further improved the correlation for the stable region to R = 0.691 and for the global region to R = 0.274. The correlation plots for Run 4 appear very similar to that of Run 3 and hence are not plotted here.

### Runs 5 to 7: post-processing

**Fig. 7:**
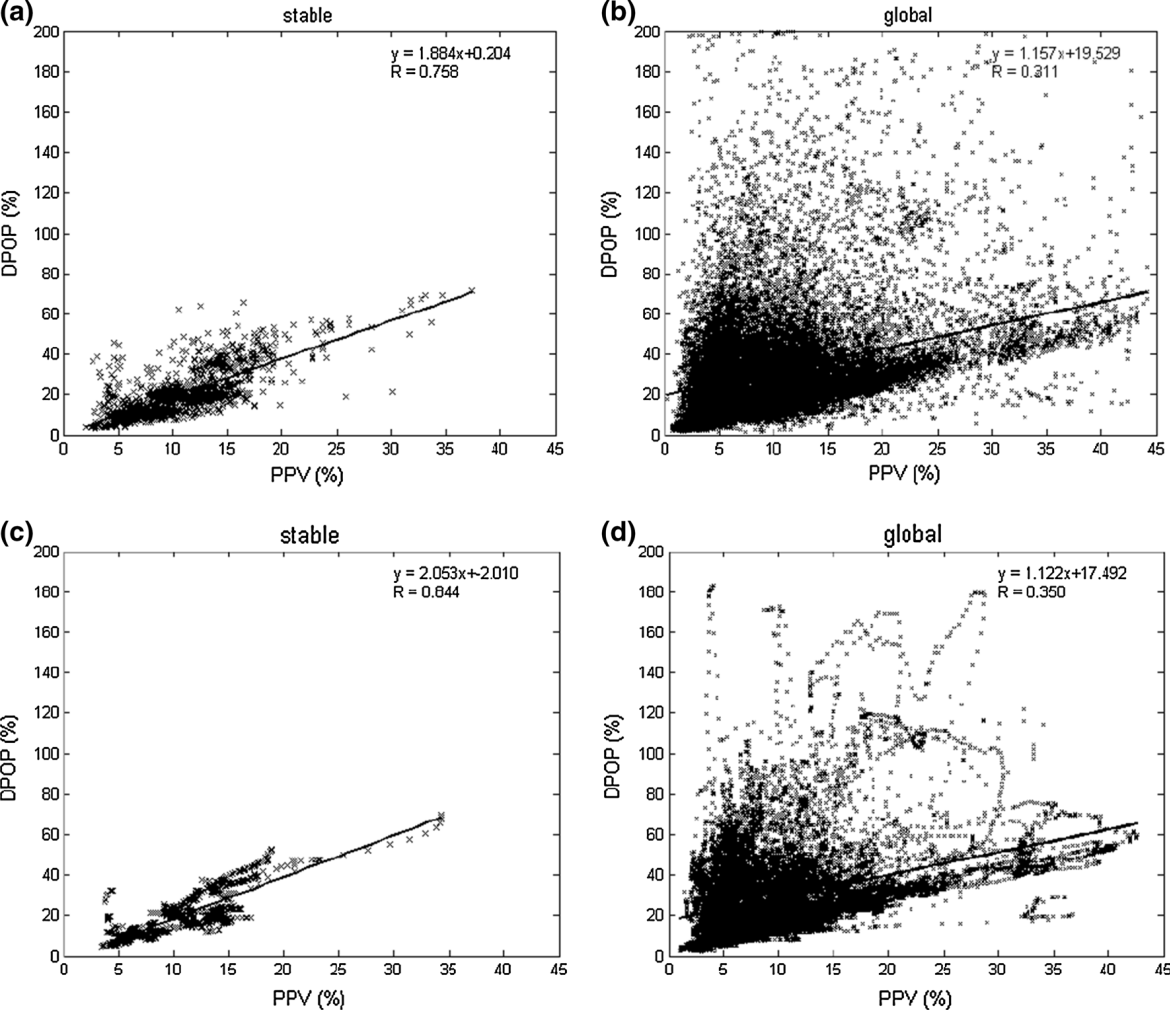
Correlation plots for Runs 5 and 7 **a** Run 5 stable region **b** Run 5 global region **c** Run 7 stable region **d** Run 7 global region

Runs 1 to 4 aimed to improve the incoming pleth signal to the algorithm in order to optimize the detection of fiducial points. These runs calculated the DPOP value within the analysis window and reported the value directly (i.e. without post-processing). This calculation was performed every 5 s and the reported DPOP value updated at the same time. The following three runs include steps to post-process this ‘instantaneous’ DPOP value in order to provide a more robust parameter. Run 5 and 6 averaged the resulting instantaneous DPOP values, first over 15 s (3 values) then 60 s (12 values). The simple mean of the data within these ‘smoothing windows’ was calculated in each case and used in the correlation plots. The results for the stable signal set increased the previous ‘raw’ Run 4 value of R = 0.691 to 0.758 (Run 5) and then 0.817 (Run 6). This further improves to 0.844 for Run 7 which employs both a 120 s smoothing window length and a percentile averaging method. The percentile averaging method only averages the data within the 25 to 75 % percentiles of the instantaneous values contained within the smoothing window. Hence for the 120 s smoothing window, 24 instantaneous values are obtained (every 5 s reported value) of which the lowest 6 and highest 6 values are removed and the mean taken of the remaining middle 12 values. This method provides both robust outlier removal and smoothing of the data. There are also successive small improvement gains in the global data results with R’s of 0.311, 0.326 and 0.350 for Runs 5 to 7 respectively. The correlation plots for post-processing Runs 5 and 7 are provided in Fig. 7. Comparing the figures we can see an obvious tightening up of the data around the best fit line for both data sets as the character of the smoothing window was changed as described.

### Runs 8 and 9: further post-processing

Without manual data selection, the pleth signal itself appears to be very vulnerable to measurement-based signal interference and movement-related baseline shifts. We limited the instantaneous DPOP values calculated by the analysis window to 70 %. Any values over 70 % were set to invalid values and not used in the calculation. We did this because manual inspection of our data, and reference to the literature for mechanically ventilated patients, revealed that all good quality sections of data were commensurate with DPOP values <70 %. In addition to this we further increased the integrity of the averaging process described for Run 7 by setting the requirement that at least 18 valid instantaneous DPOPs must be present within the 24 value smoothing window buffer; otherwise a value is not calculated. Adding these conditions to the algorithm in Run 8 resulted in a slight fall in the reported R for the stable region to 0.826 but a marked increase in the global region correlation coefficient to 0.468.

**Fig. 8:**
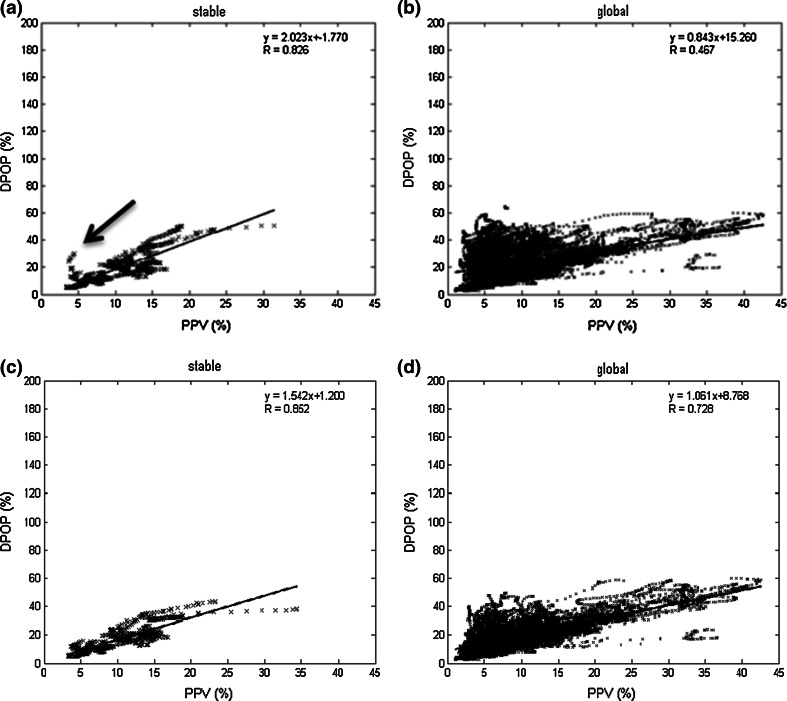
Correlation plots for Runs 9 and 10 **a** Run 9 Stable region **b** Run 9 global region** c** Run 10 stable region** d** Run 10 global region

A number of further post-processing steps were added to the algorithm in Run 9 in order to make it robust for general use (i.e. not specific to these data reported here). These are common to many pulse oximetry and other device algorithms. These included holding values (for up to 30 s) when less than 18 valid DPOPs are present within the 24 value smoothing window buffer; adding a IIR filter to the reported value; and withholding instantaneous DPOP values from the averaging process when certain internal flags were triggered, including notifications of arrhythmia, gain changes, heart rate out of range, pulse amplitude out of range and signal too noisy. Because these scenarios seldom appear in our data set, the correlation coefficient for Run 9 did not change for either data set through the addition of these extra code modules. However, in practice, these modules should always be included. The stable and global plots for Run 9 are given in Fig. 8a, b. (These are very similar to the corresponding Run 8 plots.)

### Run 10: correcting for low perfusion

In this final run, a new module was added with the specific aim of correcting the DPOP value when the pleth signal exhibited low perfusion. Low perfusion was defined when pulse amplitude values occurred at less than 3 % of the pleth baseline value. In the method, the DPOP values are corrected as follows:

2 $$\text{DPOP}_{\text{b}} = \left( {1 - \left( { - 0.8/3 \times \text{PMod} + 0.8} \right)} \right) \times \, \text{DPOP}_{\text{a}}$$

where DPOP_b_ is the corrected value, DPOP_a_ is the original value and PMod is the perfusion index [24]. The addition of this low perfusion code module improved the stable region results from R = 0.826 to 0.852. Further, a large improvement in the global region results was exhibited: from R = 0.467 to 0.728. The stable and global plots for Run 10 are given in Fig. 8c, d. This distinct improvement in the global result is also obvious in the overview results plot of Fig. 5.

## Discussion

A series of runs was conducted with increasingly sophisticated signal processing elements within an algorithm for the computation of DPOP. Through increasingly advanced pre-processing and post-processing techniques, the correlation coefficient between DPOP and PPV improved from a baseline value of R = 0.347 to R = 0.852 for the stable data set, and, correspondingly, R = 0.225 to R = 0.728 for the more challenging global data set. Early gains in performance were achieved for the stable data set using relatively straightforward algorithm improvements. However, the more challenging global data set incorporating all collected signals required significant additional algorithm improvements, including a correction for low perfusion values, before similar gains were realized.

The R values found in the present study for the final algorithm match well with many of the results reported in the literature for both OR and ICU data [3–10, 12, 13]. Many of these studies describe the algorithm employed for the computation of DPOP, all of which employ a degree of manual manipulation of the data with some attempting to automate the process to some degree. In addition, some studies plot a single averaged data point per subject, and often this may be averaged over a few hand-picked respiratory cycles, while others attempt much longer term averaging schemes. (We have considered this effect in other work [23]). This variation in method may account for much of the variability in reported values. Many research groups also cite the pre-processed nature of the pleth with which they have worked as an extra impediment to producing optimal results, as they do not have access to the raw (unfiltered) signal used by the pulse oximeter device [4, 14, 16, 17]. A full account of the various attempts to develop a DPOP algorithm is given in the review by Addison [24]. The algorithm we have described here uses the raw pleth signal acquired at the pulse oximeter probe and is fully automated, in that it must process all signals detected by the probe (i.e. all signal characteristics encountered in practice).

During our algorithm development work we continually compared the algorithm value of DPOP against manually derived values from the raw pleth waveform. However, the ultimate goal is to provide a non-invasive surrogate parameter for PPV which adds considerable complexity to the task. The detrimental effect of variable and/or low perfusion levels on methods to extract respiratory modulation information from the pleth has received attention in the literature [8, 10, 18–20] and, in fact, some groups have cited low perfusion as a criterion for excluding the data from analysis [3, 5, 9]. Although the pulse pressure waveform and pulsatile pleth waveform resemble each other [7], additional complex, nonlinear pressure-mechanical coupling between the blood fluid column, vessel walls and the body tissue matrix at the pulse oximeter sensor site separates the physiological processes giving rise to the two signals [21, 22]. The correction for DPOP accounts for the relative nonlinear changes in these two signals that occur at low perfusion which are driven by these complex physiological processes. The correction employed in this study provided a marked performance improvement for the global data set from Run 9 to Run 10 (from R = 0.467 to 0.728). This is particularly dramatic given the sophistication already incorporated within the algorithm by Run 9 achieved using a toolbox of signal processing techniques specific to the extraction of respiratory modulations from the pleth [11]. A small improvement was also achieved for the stable region from R = 0.826 to 0.852 due to the correction.

In conclusion, an automated algorithm for the determination of a robust automated DPOP parameter has been developed where the systematic gains in performance achieved by adding increasingly sophisticated signal processing elements to it have been demonstrated. Marked gains were achieved using relatively simple algorithm enhancements for the stable region data, but significant additional algorithm enhancements, including a correction for low perfusion values, were required for similar gains when considering the whole data set.
